# Role of gut microbiota and bacterial translocation in acute intestinal injury and mortality in patients admitted in ICU for septic shock

**DOI:** 10.3389/fcimb.2023.1330900

**Published:** 2023-12-18

**Authors:** Chloé Magnan, Thomas Lancry, Florian Salipante, Rémi Trusson, Catherine Dunyach-Remy, Claire Roger, Jean-Yves Lefrant, Pablo Massanet, Jean-Philippe Lavigne

**Affiliations:** ^1^Bacterial Virulence and Chronic Infection (VBIC), INSERM U1047, Univ Montpellier, Department of Microbiology and Hospital Hygiene, Platform MICRO&BIO, University Hospital Center (CHU) Nîmes, Nîmes, France; ^2^UR-UM103 UMAGINE, Univ Montpellier, Division of Anesthesia Critical Care, Pain and Emergency Medicine, CHU Nîmes, Nîmes, France; ^3^Department of Biostastistics, Epidemiology, Public Health and Innovation in Methodology, Univ Montpellier, CHU Nîmes, Nîmes, France

**Keywords:** acute intestinal injury, bacterial translocation, evolution, gut microbiota, metagenome, septic shock

## Abstract

**Introduction:**

Sepsis is a life-threatening organ dysfunction with high mortality rate. The gut origin hypothesis of multiple organ dysfunction syndrome relates to loss of gut barrier function and the ensuing bacterial translocation. The aim of this study was to describe the evolution of gut microbiota in a cohort of septic shock patients over seven days and the potential link between gut microbiota and bacterial translocation.

**Methods:**

Sixty consecutive adult patients hospitalized for septic shock in intensive care units (ICU) were prospectively enrolled. Non-inclusion criteria included patients with recent or scheduled digestive surgery, having taken laxatives, pre- or probiotic in the previous seven days, a progressive digestive neoplasia, digestive lymphoma, chronic inflammatory bowel disease, moribund patient, and pregnant and lactating patients. The primary objective was to evaluate the evolution of bacterial diversity and richness of gut microbiota during seven days in septic shock. Epidemiological, clinical and biological data were gathered over seven days. Gut microbiota was analyzed through a metagenomic approach. 100 healthy controls were selected among healthy blood donors for reference basal 16S rDNA values.

**Results:**

Significantly lower bacterial diversity and richness was observed in gut microbiota of patients at Day 7 compared with Day 0 (p<0.01). SOFA score at Day 0, Acute Gastrointestinal Injury (AGI) local grade, septic shock origin and bacterial translocation had an impact on alpha diversity. A large increase in Enterococcus genus was observed at Day 7 with a decrease in Enterobacterales, Clostridiales, Bifidobacterium and other butyrate-producing bacteria.

**Discussion:**

This study shows the importance of bacterial translocation during AGI in septic shock patients. This bacterial translocation decreases during hospitalization in ICUs in parallel to the decrease of microbiota diversity. This work highlights the role of gut microbiota and bacterial translocation during septic shock.

## 1 Introduction

Septic shock is the cause of 10 to 30% admission to intensive care units (ICUs) with mortality rates ranging from 35 to 40% (Sakr et al., 2018; Vincent et al., 2018; Vincent et al., 2019). The symptomatology is dominated by the presence of organ failures in which the intestine plays a major role (Fiddian-Green, 1988). Impaired perfusion and oxygenation of gastrointestinal tissues is classically reported despite restoration of hemodynamic parameters and systemic oxygenation after vascular filling and administration of vasopressors (Temmesfeld-Wollbrück et al., 1998). This damage induces an acute gastrointestinal injury (AGI) that can occur very early in critical illness, with a major influence on the prognostic of critically ill patients (Mutlu et al., 2001; Reintam Blaser et al., 2013; Zhang et al., 2018). Pathophysiologic mechanisms linking gut microbiota with AGI are probably multifactorial. Proposed mechanisms mainly include alterations in permeability of intestinal mucosal, increase of the host immune system due to general inflammation and activation of antigen presenting cells (Clark and Coopersmith, 2007; Reintam Blaser et al., 2013; Zhang et al., 2018). This is especially due to the specific ICU environment such as antibiotic therapy, vasopressors, mechanical ventilation and parenteral nutrition with their associated deleterious effect on the intestinal barrier. Gut microbiota dysbiosis is a hallmark of septic shock with reduction in gut microbiota diversity in ICU patients compared to healthy controls (Zaborin et al., 2014; McDonald et al., 2016; Ojima et al., 2016; Lankelma et al., 2017; Wan et al., 2018; Yin et al., 2019; Liu et al., 2020). However, none of these studies had identified low gut bacterial diversity as an independent risk factor for mortality in ICU patients with septic shock.

During AGI, an “intestinal crosstalk” takes place between the intestinal epithelium, the intestinal immune system and the gut microbiota (Niu and Chen, 2021). In critical illness, the loss of this interrelation causes systemic manifestations due to intestinal inflammation, local gut permeability and an increased permeability favorable to bacterial translocation, representing a major cause of multiple organ dysfunction syndrome (Clark and Coopersmith, 2007). Indeed, bacterial translocation is the process in which viable and/or bacterial elements cross the gastrointestinal barrier to reach the systemic circulation, disseminating microorganisms in the body (Sandler and Douek, 2012). Principal mechanisms promoting bacterial translocation are increased permeability of the intestinal mucosal barrier, deficiencies in host immune defenses and an imbalance (dysbiosis) of the diversity of gut microbiota (Balzan et al., 2007).

The gut microbiota lives in symbiosis with the body and plays a major role in progression of diverse diseases (Schmidt et al., 2018; Shanahan et al., 2021; Siwczak et al., 2021; Chen et al., 2022; Deng et al., 2022). A diverse and balanced gut microbiota strengthens the host’s immunity to intestinal and systemic pathogens, and the dysbiosis of this microenvironment is linked to increased susceptibility to sepsis (Chen et al., 2022). Previous studies have observed that sepsis and its treatment severely affect the gut microbiota (Shimizu et al., 2011; Wolff et al., 2018; Agudelo-Ochoa et al., 2020). Dysbiosis due to pathogenic microorganisms plays a key role in the sepsis, resulting in the loss of commensal gut species (Wolff et al., 2018). Moreover, the abundance of *Enterococcus* spp. acts as a prognostic marker for patients with septic complications and an increased mortality (Shimizu et al., 2011; Agudelo-Ochoa et al., 2020). However, gut microbiota involvement in AGI is not yet known.

In this study, we aimed to describe the evolution of gut microbiota profiles and AGI in patients admitted to ICU for septic shock and to highlight the role of microbiota composition as a contributing factor on poor patient outcome.

## 2 Materials and methods

### 2.1 Study design

The present prospective single center observational study was conducted according to the Declaration of Helsinki and the French law (World Medical Association, 2013; Toulouse et al., 2023). It was approved by a national ethic committee (CCP Ouest III; registration number n°2018-A02193-53) and was registered on ClinicalTrial.gov (NCT03861325). Before inclusion, the patients or representatives were informed of the study and his(her) rights to oppose to the use of their data.

### 2.2 Population

Sixty consecutive adult patients (≥ 18 years) admitted to ICU for septic shock (Nîmes University Hospital (France)) between July 2019 and September 2020 were prospectively enrolled. Septic shock was defined according to the Third International Consensus Definitions for Sepsis and Septic Shock (Sepsis-3) (Singer et al., 2016). It corresponds to a sepsis in which underlying circulatory and cellular metabolism abnormalities were profound enough to substantially increase mortality, referred to as a state of persisting hypotension despite administration of vasopressors to maintain mean arterial pressure greater than 65 mmHg, plus elevated serum lactate >2 mmol/L despite adequate fluid resuscitation. Non-inclusion criteria were: 1) a previous or scheduled digestive surgery; 2) the use of laxatives, pre- or probiotic in the previous 7 days; 3) a progressive digestive neoplasia, digestive lymphoma, chronic inflammatory bowel disease (Crohn’s disease, etc.); 4) moribund patient, or patients with a care withdrawal or withholding decision; 5) pregnant and lactating patients; 6) and patients already included in a recent interventional trial.

### 2.3 Outcomes

The patients were followed-up and we determined the early mortality corresponding to death at Day 7 and the late mortality to death at Day 28. To understand the potential impact of the evolution of gut microbiota composition, the studied population was classified in two groups: survivors (alive at Day 28) and non-survivors (died at Day 28).

For the group of healthy controls, 100 subjects were included among healthy blood donors from the French Blood Establishment (EFS, Montpellier, France). Before blood donation, the healthy controls completed a questionnaire to ensure the absence of health problem. These controls were used to determine the basal level (cut-off) of the 16S rDNA marker performed in this study.

### 2.4 Clinical, biological and therapeutic data

Baseline clinical values were recorded at ICU admission and included the following data: age, sex, weight, associated comorbidities and septic shock origin. Treatments given within 7 days were systematically recorded as well as mechanical ventilation requirement, digestive symptomatology and organ failure. Tolerance to enteral nutrition was notified at Day 3 and 7. The Simplified Acute Physiology Score II (SAPS-II) (Le Gall et al., 2005) and the Sepsis Organ Failure Assessment (SOFA) score (Singer et al., 2016) were calculated within 24 h of admission and daily recorded for 7 days, respectively. SOFA scores ≤7, between 8 and 13 and ≥14 were considered as low, moderate and high, respectively (Elke et al., 2018). The AGI local score was defined in 3 stages: AGI score 0 in patients with no symptoms and low intra-abdominal pressure; AGI score 1 in intermediate patients, with low intra-abdominal pressure but some mild symptoms; AGI score 2 in patients with severe AGI (high pressure and/or more severe symptoms). Then, a clustering method was used to categorize patients into 5 AGI grades according to the guidelines published by the European Society of Intensive Care Medicine (ESICM): AGI grade 0: normal gastrointestinal function, AGI grade I: an increased risk of developing gastrointestinal dysfunction or failure, AGI grade II: gastrointestinal dysfunction, AGI grade III: gastrointestinal failure, and AGI grade IV: marked gastrointestinal failure with severe impact on distant organ function (Reintam Blaser et al., 2012).

### 2.5 Sample collection and gut microbiota analysis

Stool and blood samples (EDTA anticoagulant tubes) were collected at ICU admission (Day 0) and at Day 7. Fecal samples were stored directly at -80°C within two hours until further processing, whereas blood samples were immediately centrifuged (1,200g; 12 min), aliquoted and stored at -80°C.

Stool DNA extractions were performed from 250 mg of fecal material using the QIAcube automatic extractor (Qiagen, Courtaboeuf, France) with the DNeasy® PowerSoil Pro® kit (Qiagen, Courtaboeuf, France) according to the manufacturer’s recommendations. A minimum volume of 50 μL at a minimum concentration of 2.5 ng/μL, measured by the QUBIT® 3.0 fluorometric (Thermo Fisher Scientific Waltham, MA, USA) with the QuantiFluor dsDNA system® kit from the same supplier, was used. Negative extraction controls were performed using sterile water for each extraction run.

Metabarcoding analysis of DNA extracts from stool samples was performed by next-generation sequencing in collaboration with Genoscreen© company (Lille, France). The 16S rDNA genes of the hypervariable V3-V4 regions were amplified for the amplicon libraries preparation according to the Metabiote® protocol of Genoscreen©. Positive controls (artificial bacterial community composed of 15 bacterial strains and 2 archaeal strains) and negative controls were also integrated. Library sequencing was performed on a MiSeq run (Illumina Inc., San Diego, CA, USA) 2x250 base pairs chemistry. After validation of a quality control of the obtained sequences, demultiplexing was performed by CASAVA (Illumina®, Paris, France) software using the PERL script ConfigureBclToFactq. A quality filter (pre-processing) and reassembly of the reads using the FLASH tool (Magoc and Salzberg, 2011) were carried out according to the parameters optimized by the company Genoscreen©. Metabiote® Online v2.0 protocol was partially based on the QIIME v 1.9.1 software (Caporaso et al., 2010). After the pre-processing steps, the full-length 16S rDNA sequences go through a step where chimeric sequences are detected and eliminated (in-house method based on the use of Usearch 6.1). Next, a clustering step was performed to group similar sequences with a defined nucleic identity threshold (97% identity for genus-level affiliation on the targeted region of the 16S rDNA gene) with Uclust v1.2.22q (Edgar, 2010) using an open reference and full linkage operational taxonomic units (OTU) creation process, ultimately creating groups of sequences or OTUs. The most abundant sequence in each OTU was then considered the reference sequence of its OTU and was taxonomically compared to a reference database (Greengenes database, version 13_8; www.greengenes.gov) using the RDP classifier v2.2 method, a naive Bayesian classifier that provided taxonomic assignments from a domain to a genus, with confidence estimates for each assignment (Wang et al., 2007). OTU rarefaction curves were calculated to ensure satisfactory sequencing effort to describe the microbial diversity of each sample.

### 2.6 Bacterial translocation

The bacterial translocation was evaluated by the quantification of 16S rDNA at Day 0 and Day 7. DNA was extracted from plasma samples with the QIAcube® automatic extractor (Qiagen, Courtaboeuf, France), from 200 μL of plasma using the QIAamp MinElute ccfDNA® kit (Qiagen, Courtaboeuf, France) according to the manufacturer’s instructions. DNA was eluted in a final volume of 80 μL. A negative extraction control was systematically extracted in parallel. Real-time qPCR was performed in Taqman technique using the Light cycler 480 II® thermal cycler (Roche diagnostics) and the LC Fast Start DNA MasterPLUS HybProbe® master mix (Roche diagnostics) in a volume of 20 μL, in 96-well plates. Primers and “Taqman” probe used targeted the V5 hypervariable region of the 16S gene as previously described by (Kramski et al., 2011). Absolute quantification analysis was performed using LightCycler® 480 Software (Roche diagnostics), version 1.5, based on a standard curve created from serial dilutions using the provided synthetic DNA. The result was expressed as a Crossing point (Cp). The Cp corresponds to the cycle of the PCR where the detection of fluorescence bends exponentially. This Cp was converted to copies/μL using the standard curve. All experiments were performed in triplicate.

### 2.7 Statistical analysis

As this work corresponded to a pilot study, no sample size calculation was performed. The statistician (F.S.) considered that 60 patients was sufficient to analyze data considering the published studies (Shimizu et al., 2011; Schmidt et al., 2018; Wolff et al., 2018; Agudelo-Ochoa et al., 2020; Shanahan et al., 2021; Siwczak et al., 2021; Chen et al., 2022; Deng et al., 2022). Statistical analysis was performed with R software version 4.1.0. The clustering algorithm to classify patients in 3 AGI grades (absence of grade 0 and low number of patients in grade IV that were integrated in a grade III-IV) over seven days was established as follows: for each level, a reference profile (centroid) was determined: group 0 took the score AGI value 0 at each time, group 1, the value 1 at each time and group 2, the value 2 at each time. Then, Euclidean distances were calculated between AGI profiles of each patient and the reference profiles of the groups so that each patient was assigned to their closest group. Phyloseq and vegan R packages were used for Metagenomics analyses. The distribution of OTUs and the composition of microbial communities were analyzed by determining their relative abundance at phylum and genus levels. α-diversity represented by Shannon and Chao-1 scores while β-diversity was assessed using Principal Coordinate Analysis (PCA) with Bray-Curtis dissimilarity indices. PCA was also used to show discrimination between groups according to a selection of differentially abundant bacteria. The association between gut microbiota composition and late mortality (survivors vs non-survivors) was assessed by Mann-Whitney test. The area under the receiver operating characteristic (ROC) curve (AUC) was used to illustrate the discriminatory power of bacteria according to the survivors at day 28.

## 3 Results

### 3.1 Characteristics of population

**Table 1** and **Figure S1** show patient baseline characteristics. At day 28, 16 patients have died (late mortality = 26.7%). Among them, 6 patients died within the first week (early mortality = 10.0%). There was no statistical significant difference between survivors and non-survivors at Day 7 and Day 28 (late mortality), except a higher SAPS II score in non-survivors group (62.8 ± 19 versus 51.5 ± 16.8, p=0.0012).

**Table 1:** Patient baseline characteristics.

Characteristics	Total (n=60)	Non-survivors (n=16)	Survivors (n=44)	p-value
Age, years	75 (67-80)	79.5 (79-80)	74.5 (67.3-80.8)	0.21
Sex ratio, Female/Male	25/35	6/10	19/25	1
BMI, kg/m^2^	27.3 (23.4-30.1)	27.3 (23.9-28.3)	27.3 (23.4-30.8)	0.56
**SOFA score**	8.5 (7-10)	10 (8.5-10)	8 (7-10)	0.39
**AGI grade 0**	0	0	0	
** I**	5	0	5	
** II**	34	13	21	0.12
** III**	14	2	12	
** IV**	3	1	2	
Comorbidities				
Coronaropathy	13 (21.7)	6 (37.5)	7 (15.9)	0.09
Heart insufficiency	7 (11.7)	4 (25.0)	3 (6.8)	0.07
Arterial hypertension	41 (68.3)	11 (68.8)	30 (68.2)	1
Diabetes mellitus	20 (33.3)	3 (18.7)	17 (38.6)	0.22
Chronic renal failure	11 (18.3)	3 (18.7)	8 (18.1)	1
Alcoholism	4 (6.7)	2 (12.5)	2 (4.5)	0.29
Hepatic cirrhosis Child A-B	1 (1.7)	1 (6.3)	0 (0)	0.27
COPD	7 (11.7)	2 (12.5)	5 (11.3)	1
Cancer solid/hemopathy	4 (6.7)	1 (6.3)	3 (6.8)	1
Sources of infection				
Pulmonary	21 (35.0)	8 (50.0)	13 (29.5)	0.22
Urinary	16 (26.7)	1 (6.3)	15 (34.1)	0.06
Digestive	8 (13.3)	1 (6.3)	7 (15.9)	0.67
Skin and Soft Tissue Infections	5 (8.3)	2 (12.5)	3 (6.8)	0.60
Other	7 (11.7)	2 (12.5)	5 (11.3)	1
Unknown origin	3 (5.0)	2 (12.5)	1 (2.3)	0.17
**Previous hospitalization <3months**	21 (35.0)	5 (31.3)	16 (36.4)	0.77
**Previous antibiotherapy <3months**	14 (23.3)	2 (12.5)	12 (27.3)	0.31
Mortality				
Day 7	6 (10)	6 (37.5)	0 (0)	not applicable
Day 28	16 (26.7)	16	0	not applicable

BMI, Body Mass Index; COPD, Chronic Obstructive Pulmonary Disease.

### 3.2 Evolution of community richness, diversity and structure of the gut microbiota in septic shock patients

Gut microbiota data was available in all 60 patients at ICU admission (Day 0) and at Day 7. The assembly parameter applied at 97% nucleic identity allowed the assembly of full-length 16S rDNA sequences of 85.45% on average and representing a total of 2,865,340 reads and 29,847 full-length 16S rDNA sequences per sample on average.

**Figure 1:**
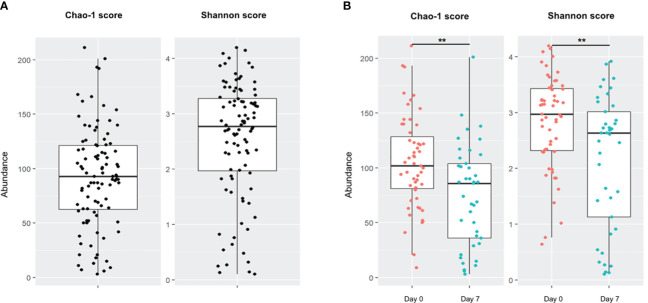
Alpha diversity of patient population. Boxes represent the IQR between the first and third quartiles; the horizontal line represents the median. **(A)** Chao-1 and Shannon scores on global population. **(B)** Comparison of Chao-1 and Shannon scores on global population between Day 0 and Day 7. (**, p <0.01).

Community richness and diversity were estimated by Chao-1 and Shannon scores, respectively. In all population samples combined (Day 0 and Day 7), the median Shannon score was 2.78 [1.97-3.27] and a median Chao-1 score was 93 [62.50-121.25] (**Figure 1A**). There was a high disparity in sample diversity with a Shannon score varying from 0.10 to 4.19. Sample diversity and richness at Day 7 were significantly decreased compared to those on Day 0, estimated by Shannon score (2.97 at Day 0 vs 2.63 at Day 7; p=0.0045) and Chao-1 score (102 at Day 0 vs 86 at Day 7; p=0.0029) (**Figure 1B**). The alpha diversity evolution between D0 and D7 showed a decrease of the Shannon score (-0.75 [-1.90;-0.02]) and Chao-1 score (-28 [-62.00; 1]).

Finally, the PCA divided gut microbiota of patients into two homogeneous groups. The microbiota had lower variability at Day 0, with much higher variability at Day 7 (**Figure S2**).

### 3.3 Taxonomic composition of the gut microbiota and their evolution

The evolution of taxonomic composition of the gut microbiota of patients in septic shock are detailed in **Figure S3**, and **Tables S1**, **S2**.

At the phylum level, the Firmicutes and Bacteroidota were increased between Day 0 and Day 7 (Mean: 72.58% ±23.17 vs 79.61 ±21.93, p=0.043; 5.96 ±9.95 vs 9.8 ±15.82, p=0.929, respectively), whereas the Proteobacteria and Actinobacteriota were significantly decreased (12.21 ±18.57 vs 4.18 ±0.85, p=0.0009; 8.88 ±14.81 vs 5.9 ±11.96, p=0.014) (**Table S1**). The increase of Firmicutes was mainly due to a strong abundance of *Enterococcus* and, to a lesser degree, of *Christinensenella* and *Staphylococcus*. In contrast, among the Clostridiales order of Firmicutes, some OTUs within the Tissierellacea family (e.g., *Finegoldia*, *Peptoniphilus*, *1855D* genera), Lachnospiraceae (e.g., *Blautia, Lachnospira, Coprococcus, Clostridium, Dorea, Roseburia* genera), and Ruminococcaceae (e.g, *Ruminococcus*, *Butyricicoccus*) were significantly decreased at Day 7 (p<0.05). The same trend was observed for *Sarcina, Dialister* and *Anaerofustis*. Among Proteobacteria and Actinobacteriota phyla, some Enterobacterales (e.g., *Escherichia*, *Shigella* genera) and *Bifidobacterium* were also in a significant lower abundance at Day 7 compared to Day 0 (p=0.041). Finally, two Bacteroidales (*Parabacteroides* and *Alistipes*) were significantly abundant at Day 7 compared to Day 0 (p=0.019 and p=0.045, respectively) (**Table S2****).**

A selection of bacteria genera, in particular *Enterococcus*, *Christinensenella*, *Staphylococcus, Parabacteroides* and *Alistipes* discriminated fecal samples at Day 7 compared to Day 0 on the PCA graphic (**Figure S4**).

### 3.4 Relationship between clinical indicators and evolution of gut microbiota

**Figure 2:**
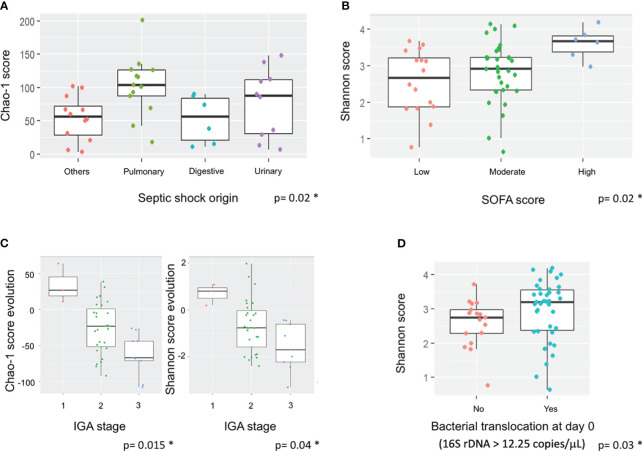
Impact of septic shock origin, SOFA score, AGI grades and bacterial translocation on alpha diversity and its evolution. Boxes represent the IQR between the first and third quartiles; the horizontal line represents the median. Statistical significance was tested using the Mann-Whitney test. **(A)** Impact of septic shock origin on Chao-1 score at Day 7. **(B)** Impact of SOFA score at Day 0 on Shannon score at admission. **(C)** Impact of IGA grade change in richness and diversity. **(D)** Impact of BT at admission on Shannon score at admission. * corresponds to significant statistical difference with the p value.

The impact of different clinical parameters was evaluated on alpha diversity and its evolution over the seven days (**Table 2**). At Day 7, Chao-1 score (richness) was higher in patients with a pulmonary origin of sepsis compared to the others (p=0.02) (**Figure 2A**). There was a higher diversity of the microbiota at admission in patients with high SOFA score (p=0.02) (**Figure 2B**) associated with a significantly high Shannon score (3.68 at Day 0 vs 2.68 at Day 7; p=0.017). Late mortality and SAPS II score did not have any impact on microbiota diversity in our population, despite the gut microbiota diversity of survivors (at Day 28) being lower compared to the diversity of the non-survivors (**Table 2**).

**Table 2:** Impact of clinical parameters on alpha diversity (Shannon and Chao-1 score) and its evolution between Day 0 and Day 7.

Parameters		Alpha diversity				Alpha diversity evolution	
		Day 0		Day 7			
		Shannon score median	Chao-1 score median	Shannon score median	Chao-1 score median	Shannon score median	Chao-1 score median
**Mortality** (at Day 28)	Survivor	2.66	87	2.46	60	-0.765	-29
	Non-Survivor	3.16	122	3.02	101	-0.581	-26
	*p*-value	***0.03***	***0.001***	*0.08*	*0.08*	*0.99*	*0.72*
**Septic shock origin**	Pulmonary	3.2	118	3.2	104	-0.331	-18.4
	Digestive	1.93	73	2.03	56	-0.533	-27
	*p*-value	*0.08*	*0.10*	*0.07*	***0.02***	*0.33*	*0.71*
**AGI grade**	I	2.99	104	2.63	90	0.806	27
	II	2.91	96	2.71	87	-0.759	-23
	III-IV	3.17	106	1.42	38	-1.7	-67
	*p*-value	*0.69*	*0.86*	*0.10*	*0.08*	***0.015***	***0.004***
**AGI score**	0	2.85	84.5	3.02	100	0.238	14
	1	3.21	106	2.55	87	-0.961	-27.5
	2	3.12	105	1.96	63	-1.1	-44
	*p*-value	*0.34*	*0.31*	*0.21*	*0.23*	***0.007***	***0.009***
**SOFA score**	Low	2.68	95	2.03	49	-0.664	-44
	Moderate	2.92	102	2.61	86	-0.772	-23
	High	3.68	149	2.9	93.5	-0.666	-41
	*p*-value	***0.02***	*0.11*	*0.36*	*0.41*	*0.924*	*0.521*
**IGS II**	Low	2.87	102	2.49	52	-0.581	-38
	Moderate	2.96	110	2.62	87	-0.773	-28
	High	3.22	100	2.85	94	-0.527	-24.5
	*p*-value	*0.37*	*0.56*	*0.66*	*0.37*	*0.935*	*0.738*

AGI, acute gastrointestinal injury. Statistical significance was tested using the Mann-Whitney-Wilcoxon test. Bold values represent statistically significant differences (p<0.05).

An association between some genera and AGI grade was observed. At admission, *Claocibacillus* was more present in AGI grade I and II (p=0.003, q=0.713), *Oisenella* and *Parabacteroides* in AGI grade I (p=0.017, q=1, respectively) and *Gallicola* in AGI grade III-IV (p=0.022, q=1). At Day 7, *Butyricicoccus* was decreased in all AGI grades (p=0.0066, q=0.859), and *Klebsiella* in AGI grade I and III-IV (p=0.0126, q=1), whereas this genus increased in AGI grade II (**Table S3**). Comparison between Day 0 and Day 7 identified some variations in the genera repartition: patients with AGI grade I presented a significant increase of *Blautia, Dialister* and *Ruminococcus* whereas at AGI grade II and III-IV, we observed a decrease of these species but also *Anaerostipes* and *Dorea.*

The association between SAPS II score and genera showed that at inclusion, the severe score of SAPS II was associated with the significant detection of *Gardnerella, Clostridium* and *Collinsella* (p=0.03, q=1). At Day 7, the moderate SAPS II score was associated with an increase of *Enterococcus*, *Alistipes*, and *Roseburia* (p<0.05, q=1) (**Table S3**).

Interestingly, a severe SOFA score was observed in presence of *Akkermansia, Blautia, Commamonas, Gardenerella, Faecalibacterium, Butyrivibrio, Parabacteroides* and *Alloscardovia*, whereas SOFA score was less severe in patients harboring *Eggerthella* in their gut microbiota (p<0.05; q=1). At Day 7, the severity of SOFA was associated with the presence of *Peptococacceae, Proteus*, and *Blautia* (p<0.04; q=1). Finally, the evolution of the genera between D0 and D7 was correlated with the significant decrease of *Akkermansia* and *Gardenella*, and an increase of *Clostridiaceae* in patients with severe SOFA score (p<0.04; q=1) (**Table S3**).

Finally, some genera were associated with the origin of septic shock. Indeed, *Streptococcus* was associated with gut origin, *Methanobrevibacter* with pulmonary origin, *Enterococcus* with gut and urine origin and *Klebsiella* with other origins (**Figure S5**).

### 3.5 Impact of AGI on gut microbiota and bacterial translocation

As the link between gut inflammation, dysbiosis and BT has been previously proposed (Mutlu et al., 2001), we determined BT by qPCR of 16S rDNA. Using control patients, a cut-off of BT corresponding to a value >12.25 copies/μL was determined. The median plasma 16S rDNA detected in the septic shock patients was 15.70 copies/μL at admission and 19.57 copies/μL at Day 7, significantly higher than in healthy controls (8.96 copies/μL) (p<0.01) (**Table 3**).

**Table 3:** Determination of bacterial translocation by the quantification (qPCR) of 16S rDNA in septic shock patients and healthy controls.

	Septic shock patients n=59		Healthy controls n=100	*p*-values
	D0	D7	D0	
**Total**	15.70 (10.71-27.06)	19.57 (11.36-30.55)	8.96 (8.14-9.86)	<0.001
**AGI score 0**	15.70 (8.59-25.08)	15.97 (8.86-3.61)	–	
**AGI score 1**	15.87 (13.19-28.05)	23.46 (17.92-25.45)	–	0.077
**AGI score 2**	19.97 (10.58-38.48)	25.59 (15.41-33.9)	–	
**Survivor at D28**	14.75 (9.90-18.55)	17.57 (10.75-28.94)	–	0.403
**Non-survivors**	16.80 (11.13-28.40)	24.89 (19.45-31.92)	–	

Statistical significance was tested using the Kruskal Wallis test (for the total) and a mixed ANOVA text (for AGI score). Bold values represent statistically significant differences (p<0.05). All data are expressed by median and IQR.

The bacterial translocation was evaluated according to the AGI score and patient mortality. Data are expressed as copies/μL.

Shannon score was significantly correlated to a high BT (>12.25 copies/μL) at admission (p=0.03) (**Figure 2D**). There was a strong association between the AGI grades and the richness and diversity of the gut microbiota of the patients. The patients with an AGI grade 3 had a significantly greater reduction of gut microbiota diversity than those with an AGI grade 2, whereas patients with an AGI grade 1 had an increase of their microbiota diversity (Shannon score; p=0.015) (**Figure 2C**). The same trends were observed with AGI scores at admission (**Table 2**).

Moreover, we observed that AGI score at admission and Day 7 also significantly influenced BT (15.7 and 15.97 for score 0 vs 19.97 and 25.59 for score 2, respectively; p=0.077).

### 3.6 Relationship between mortality and evolution of gut microbiota

The alpha diversity of the gut microbiota was significantly lower in non-survivors at admission: Shannon score (2.66 vs 3.16, p=0.03) and Chao-1 score (87 vs 122, p=0.013) (**Figure S6**; **Table 2**). However, the evolution of the alpha diversity of gut microbiota at Day 7 was not correlated with poor evolution of septic shock (p=0.08) (**Table 2**).

There was a significantly higher abundance of *Mogibacteriaceae, Robinsoniella, Klebsiella* and *Proteus* at admission in the non-survivor vs survivor groups (p<0.03, q=1) (**Table S4**). In the survivors, the gut microbiota had no variation in genera during the first 7 days (**Figure S6**). In the non-survivors, there was a significant decrease of *Escherichia* (p=0.03), and to a lesser extent, *Morganella* (p=0.029), *rc4-4* (p=0.023) and *Robinsoniella* (p=0.029) and the significant increase of *Pseudomonas* (p=0.029), *Christensenellaceae* (p=0.038) and to a lesser extent, *Enterococcus* (p=0.007), and *Actinomyces* (p=0.015) (**Figure S7**; **Table S4**).

**Figure 3:**
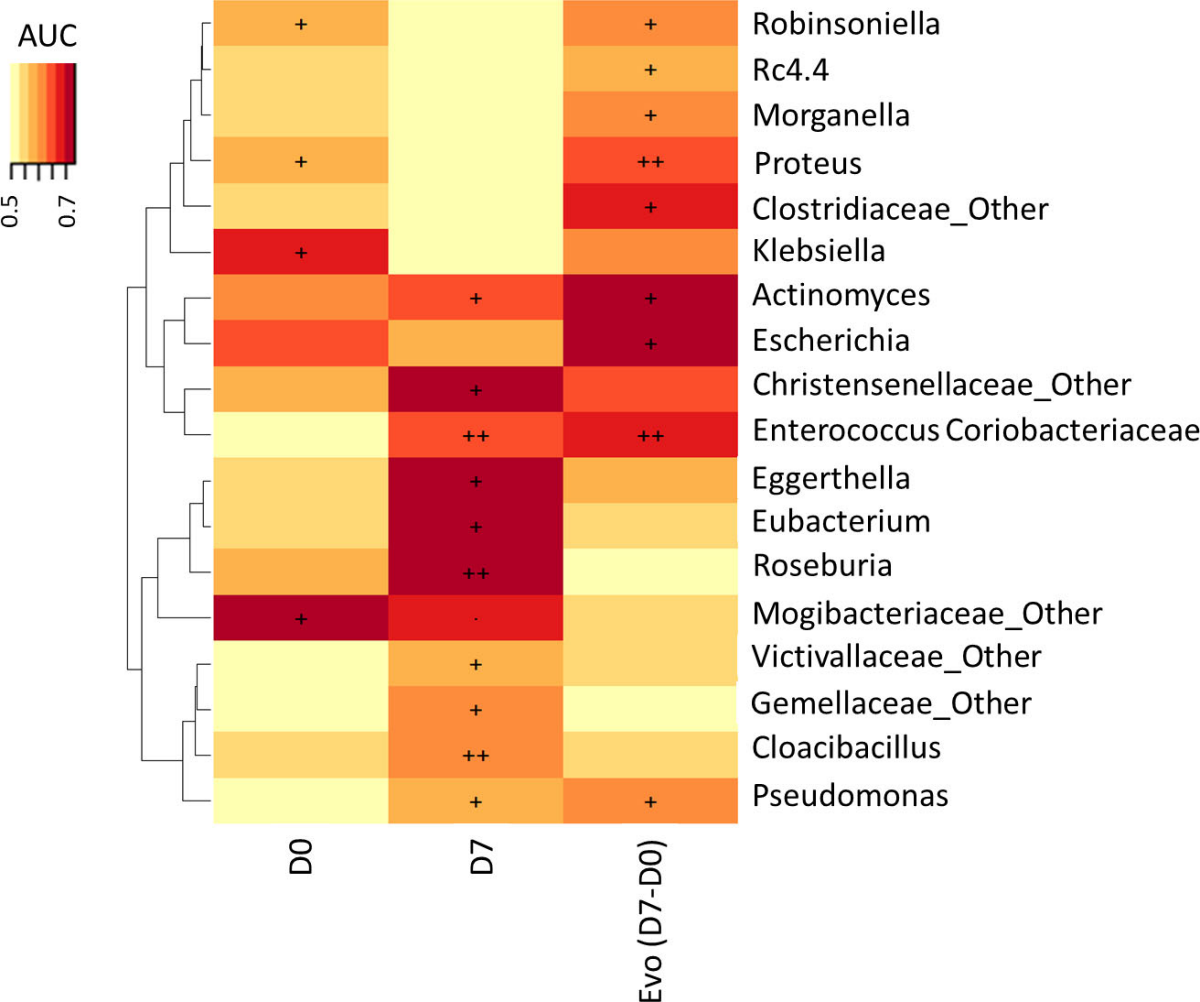
Association of vital status at Day 28 with microbiota genus levels at Day 0 and Day 7 and change between Day 0 and Day 7. The heat map of Area under the ROC Curve (AUC) between abundance at genus level and vital status at Day 28 (., p < 0.10; +, p < 0.05; ++, p < 0.01).

The heat map of Area under the ROC Curve (AUC) between relative abundance at genus level and vital status at Day 28 showed that an increase of *Enterococcus, Pseudomonas, Clostridiaceae* and *Actinomyces* and a decrease of *Proteus* and *Escherichia* were significantly associated with mortality (p<0.05; q=1) (**Figure 3**). There was no significant difference of BT at admission between survivors and non-survivors (21.9 ± 13.85 copies/μL vs 26.7 ± 45.28, respectively; p=0.403).

## 4 Discussion

The present study confirmed the decrease of richness and diversity of the gut microbiota in septic shock patients between inclusion and Day 7. It confirms an increase of Firmicutes and Bacteroidota, as previously noted (Shimizu et al., 2011; Zaborin et al., 2014; Liu et al., 2020). The increase of these phyla was directly linked to the increase of *Enterococcus*, *Christinensenella* and *Staphylococcus* among Firmicutes, and *Parabacteroides* and *Alistipes* among Bacteroidota. In parallel, there was a decrease of Proteobacteria and Actinobacteria. Among this last phyla, *Bifidobacterium* genus is an important member of the human gut bacterial population throughout life, with health benefits associated with anti-inflammatory properties (Khokhlova et al., 2012; O’Callaghan and van Sinderen, 2016) and decreased intestinal permeability (Underwood et al., 2015). *Bifidobacterium* supplementation decreases intestinal LPS levels and improves the barrier properties of the intestinal mucosa in mice (Griffiths et al., 2004; Wang et al., 2006). Indeed, in germ-free mice colonized with human gut microbiota, increased levels of *Bifidobacterium* were associated with decreased BT to the systemic circulation (Romond et al., 2008).

Several studies have shown differences in gut microbiota composition in septic shock (Shimizu et al., 2011; Wan et al., 2018; Yin et al., 2019; Agudelo-Ochoa et al., 2020; Liu et al., 2020), whereas the influence of AGI and BT elements from the gut microbiota on inflammation/infection status remains unclear (Clark and Coopersmith, 2007). Here, we clearly highlighted a gut dysbiosis and BT present in patients with a severe AGI (grade 3) (**Figure 2**; **Tables 2**, **3**). According to the AGI grade at admission, the present study highlighted a great diversity of the gut microbiota with low BT values at grade I, whereas a low diversity and high BT values were observed at grade III-IV. This finding reinforces the idea that gut inflammation could help select some phyla or genera and that gut permeability favors BT. In this context, *Oisenella* and *Parabacteroides* were preferentially detected at AGI grade I and *Gallicola* at AGI grade III-IV at admission. Interestingly, an increase of *Blautia, Dialister* and *Ruminococcus* was observed at AGI grade 1, representing species that could protect against gut inflammation. Moreover, the decrease of *Anaerostipes* and *Dorea* at AGI grade III-IV could also participate in this protection. Among these genera, *Blautia, Dialister* and *Ruminococcus* belong to short chain fatty acids (SCFA) producers of Ruminococcaceae and Lachnospiraceae families (Louis and Flint, 2017). SCFAs have anti-inflammatory, anti-cancer, and anti-oxidant properties and prevent intestinal permeability. These metabolites have previously been shown to be significantly lower in critical patients than in healthy controls (O’Keefe et al., 2011).

*Enterococcus* includes opportunistic microorganisms that enhance the virulence of pathogens (Lavigne et al., 2008), and the host immunity and inhibit overgrowth of opportunistic pathogens by producing SCFAs (Zhao et al., 2018) and bacteriocins (Brandão et al., 2010). Recently, Liu et al. described an enterotype mainly composed of *Enterococcus* in patients with sepsis associated with a lower occurrence of septic shock, speculating that *Enterococcus* could be a protective biomarker in their population (Liu et al., 2020). Interestingly, in our population, *Enterococcus* had low abundance in the gut microbiota at admission, confirming its possible protective role against septic shock. However, this genus was significantly correlated with severe SAPS II score and was significantly increased in non-survivors between Day 0 and Day 7, as previously observed (Shimizu et al., 2011; Agudelo-Ochoa et al., 2020). This result suggests that *Enterococcus* has probably been selected during hospitalization (due to the different drugs used) and corroborates the idea that this genus must be associated with a worsening prognostic marker of septic shock. We also observed that *Enterococcus* was preferentially isolated in septic shock from gut and urine origins, suggesting that the intestine reservoir is essential in the disease. Altogether, our results indicate that, while predominant *Enterococcus* may have a protective role at admission, their increase during ICU hospitalization could represent a worsening prognosis. The clear origin of the emergence and colonization of the intestine by enterococci during hospitalization must be determined to combat septic shock-related mortality. *Enterococcus* was not the only genus linked to the late mortality of the patients. *Pseudomonas, Clostridiaceae* and *Actinomyces* were also significantly increased in the gut microbiota of non-survivors, whereas *Proteus* and *Escherichia* were significantly decreased (**Figure 3**). The decrease of these two last genera can be correlated with the decrease of Proteobacteria phylum. *Pseudomonas, Clostridiaceae* and *Actinomyces* were not associated with AGI grades, and may have been acquired during hospitalization. *Pseudomonas* is a well-known hospital bacterium, particularly present in ICU, affecting immunocompromised patients (Kang et al., 2003). *Clostridiaceae* and *Actinomyces*, two intestinal commensal bacteria, were selected in the gut. They represent opportunistic bacteria that can cause hospital-acquired infection in damaged epithelia where they reside in ‘microniches’ with low oxygen, favoring anaerobic growth, especially in the deepest layers. ICU management, particularly the use of antibiotics, is the main driver of this selection. Antibiotic use often causes gastrointestinal adverse events, and is usually attributed to change in the composition and diversity of gut microbiota (Lama et al., 2019). The presence of genera identified as non-protective factors also suggests that only one genus or species in gut microbiota are frequently unable to participate alone in the gut inflammation. It is probably the addition of some genera or species that must establish networks of interspecies interconnection modifying the intestine crosstalk. Finally, BT was not significantly correlated with patient outcome, despite gut dysbiosis. We hypothesize that the determination of the BT on admission was either too late or too early in the septic shock process, explaining the higher mortality of our patients after the seventh day.

The main limitation of our study was the single center recruitment that could bias the interpretation of the results and limit its generalization. However, our population was homogenous and notably in their geographical location avoiding some ethnic variations important in gut microbiota analysis. Moreover, some results supported those obtained previously in other teams.

In conclusion, our study highlighted the importance of association between AGI, dysbiosis and BT in patients with septic shock, and reinforces the link between dysbiosis and mortality. This gut inflammation was associated with dysbiosis where potential bacteria differed significantly over time in patients with septic shock. Our results suggest that intestinal and environmental bacteria present in ICU are involved in the AGI severity and the mortality, perpetuating the chronicity of the systemic inflammation. The control of this inflammation remains an objective in the ICU management. Moreover, understanding the mechanisms between inflammation and intestinal bacteria could help to develop future therapeutic strategies in septic shock by targeting the intestinal microbiota.

## Data availability statement

The data presented in the study are deposited in the NCBI/NLM repository (Bioproject), accession number PRJNA1034825.

## Ethics statement

The studies involving humans were approved by Comité de Protection des Personnes Ouest III (France). The studies were conducted in accordance with the local legislation and institutional requirements. The participants provided their written informed consent to participate in this study.

## Author contributions

CM: Data curation, Formal analysis, Investigation, Methodology, Resources, Software, Validation, Writing – original draft. TL: Data curation, Formal analysis, Investigation, Resources, Validation, Writing – review & editing. FS: Formal analysis, Methodology, Software, Validation, Writing – review & editing. RT: Data curation, Formal analysis, Writing – review & editing. CD-R: Data curation, Formal analysis, Writing – review & editing. CR: Conceptualization, Data curation, Formal analysis, Investigation, Visualization, Writing – review & editing. J-YL: Investigation, Project administration, Supervision, Validation, Visualization, Writing – review & editing. PM: Conceptualization, Data curation, Formal analysis, Funding acquisition, Investigation, Methodology, Project administration, Supervision, Validation, Writing – review & editing. J-PL: Conceptualization, Funding acquisition, Investigation, Methodology, Project administration, Resources, Supervision, Validation, Visualization, Writing – original draft.
