# Supplementary material for: Role of gut microbiota and bacterial translocation in acute intestinal injury and mortality in patients admitted in ICU for septic shock
Source: Front Cell Infect Microbiol. 2023 Dec 18;13:1330900. doi: 10.3389/fcimb.2023.1330900 (PMC10765587; doi:10.3389/fcimb.2023.1330900)
Supplement: Supplementary file 1 [file DataSheet_1.docx]

Supplementary Material

# Supplementary Figures

**Figure S1**. Flow Chart of the study


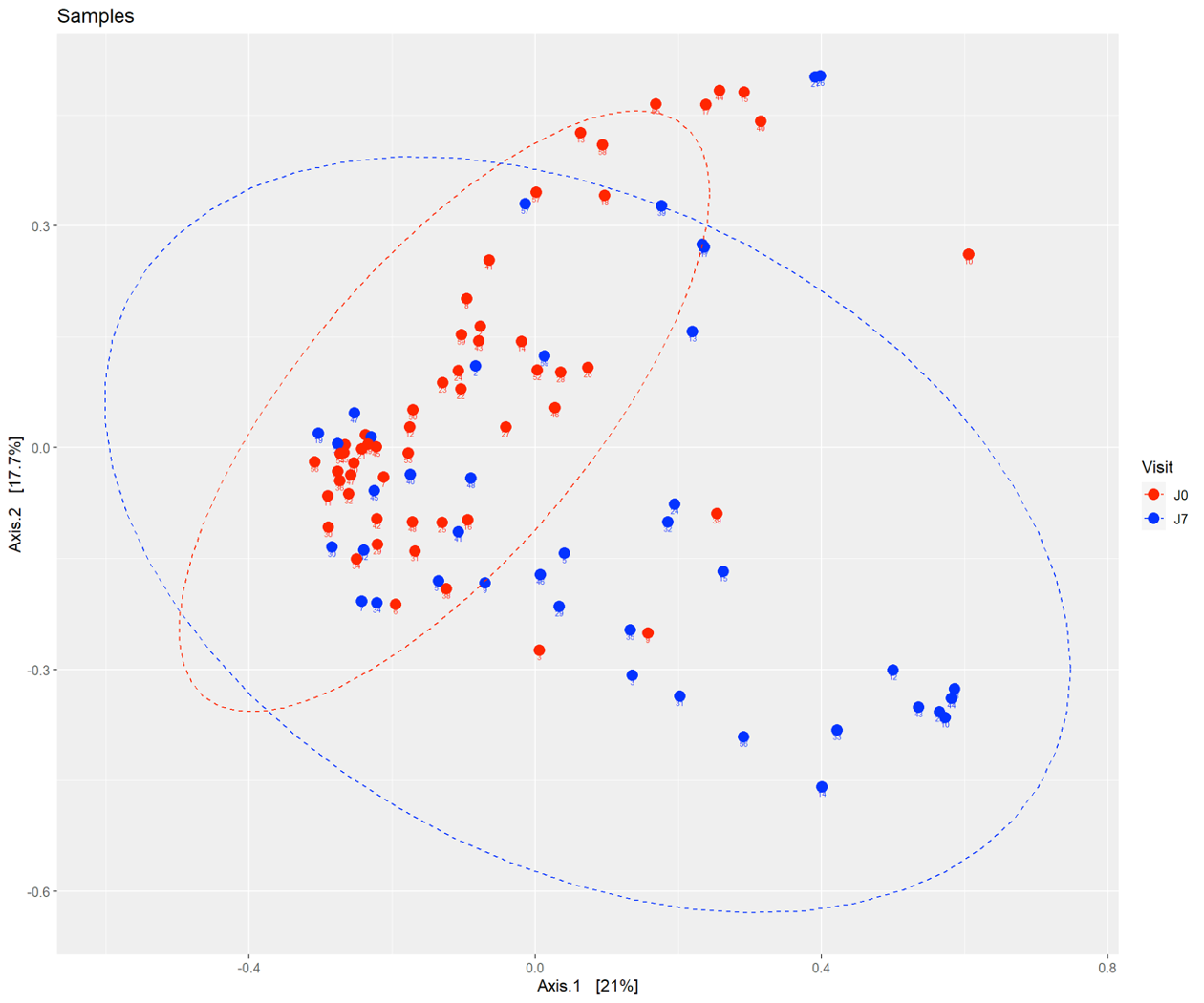


**Figure S2. Beta diversity of patient cohort.** Principal coordinate analysis (PCoA) based on the overall structure of the stool microbiota in all samples. Each data point represents an individual sample. PCoA was calculated using Bray-Curtis dissimilarity. Ellipses are at 95% confidence level. Color is indicative of the time (Day 0 in red or Day 7 in blue).


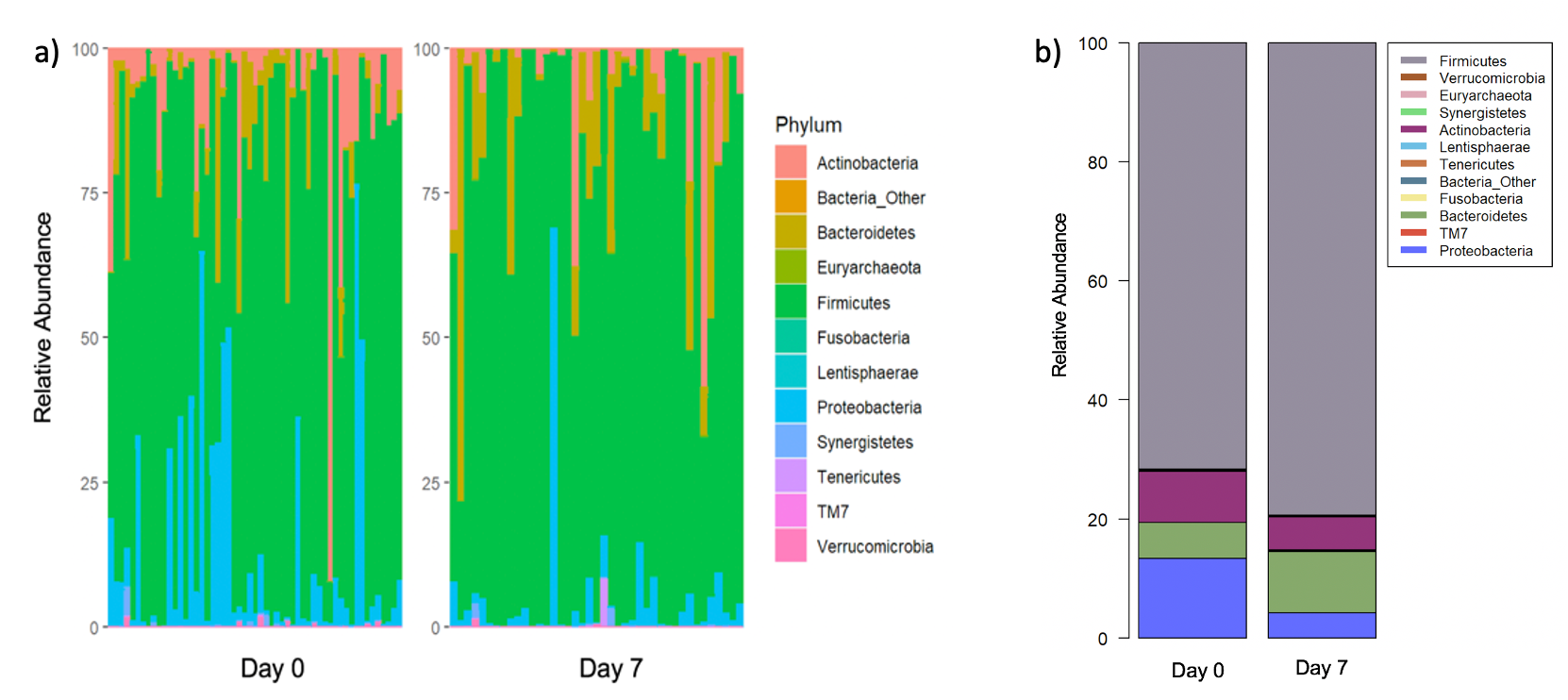


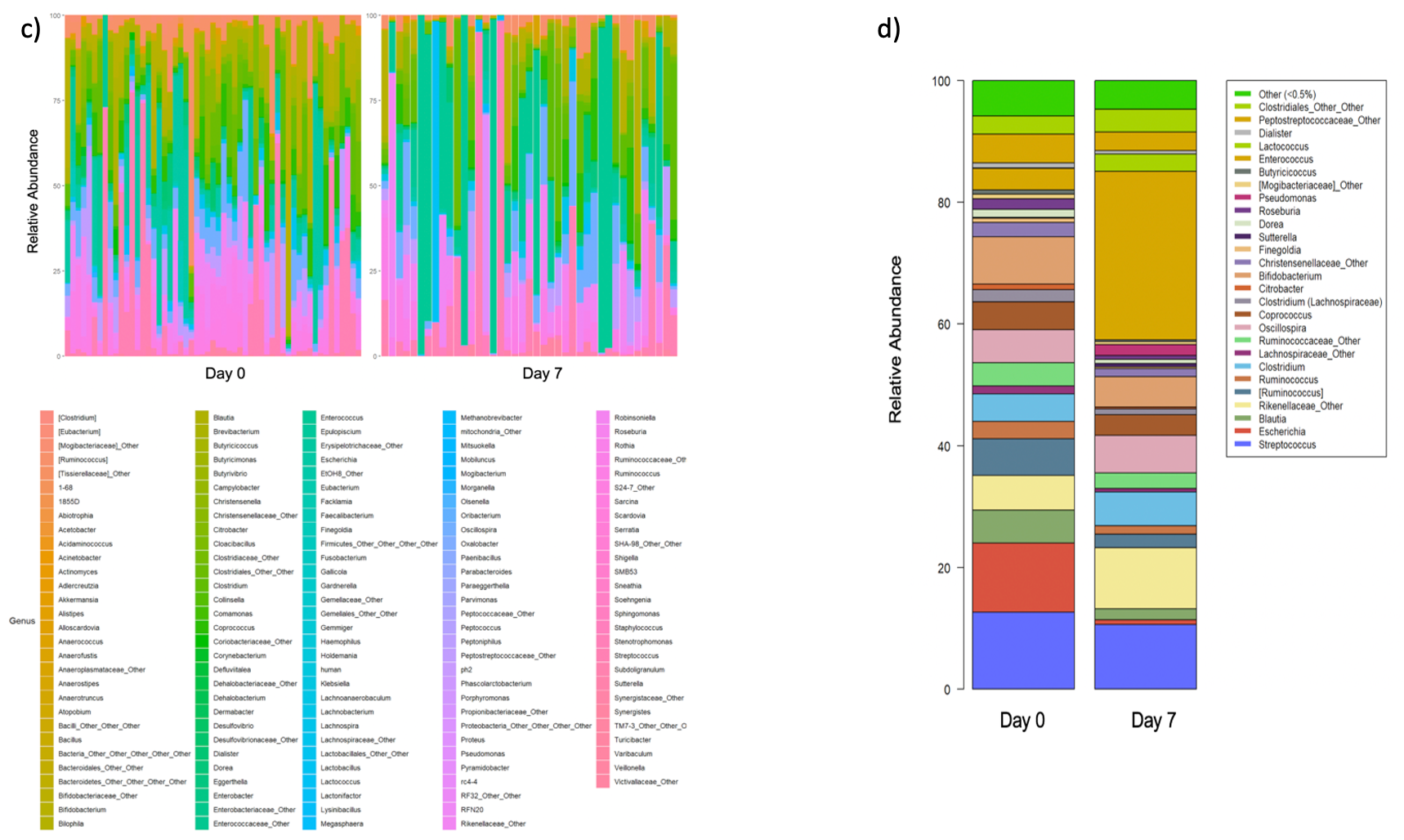


**Figure S3**. **Comparison of taxonomic composition at phylum and genus level between Day 0 and Day 7**. a) Study of all samples at phylum level; b) Sample average at phylum level; c) Study of all samples at genus level; d) Sample average at genus level.


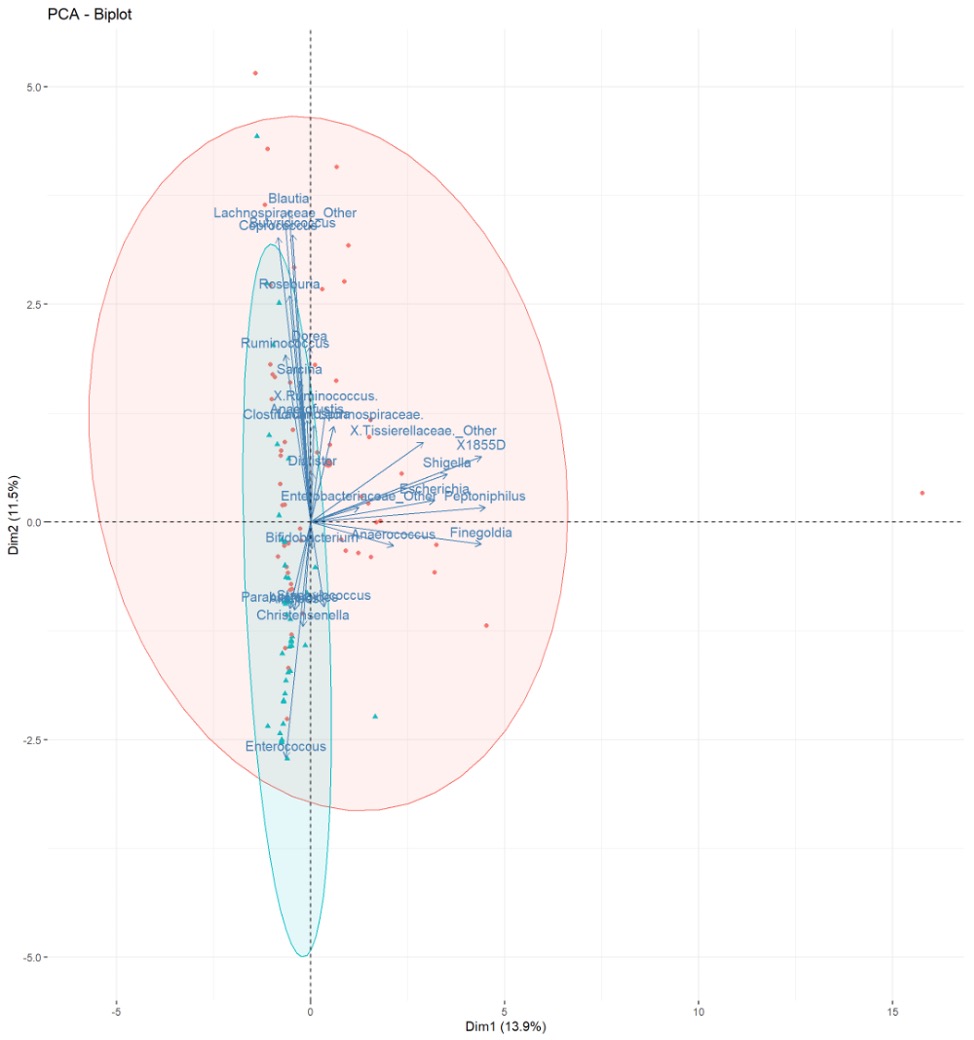


**Figure S4. Principal component analysis** **based on relative abundance of a selection of bacterial genera (the ones having a statistically significant evolution**). Each data point represents an individual sample. Color is indicative of the time (Day 0 in red and Day 7 in blue)


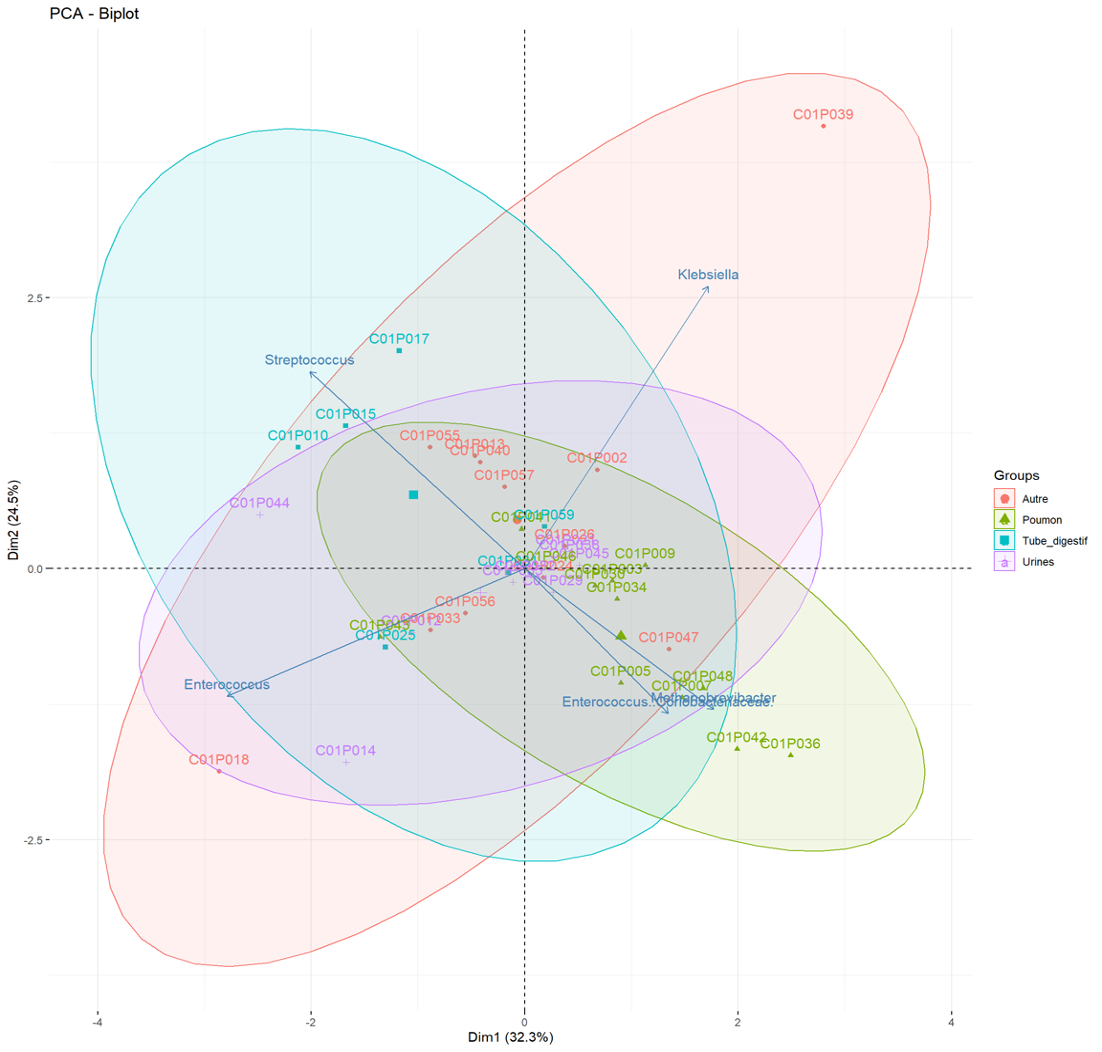


**Figure S5. Principal component analysis** **based on relative abundance of bacterial genera (those showing a statistically significant change**) **and the origin of the septic shock**. Each data point represents an individual sample. Color is indicative of the groups (gut in blue, lung in green, urine in purple and other origin in red).


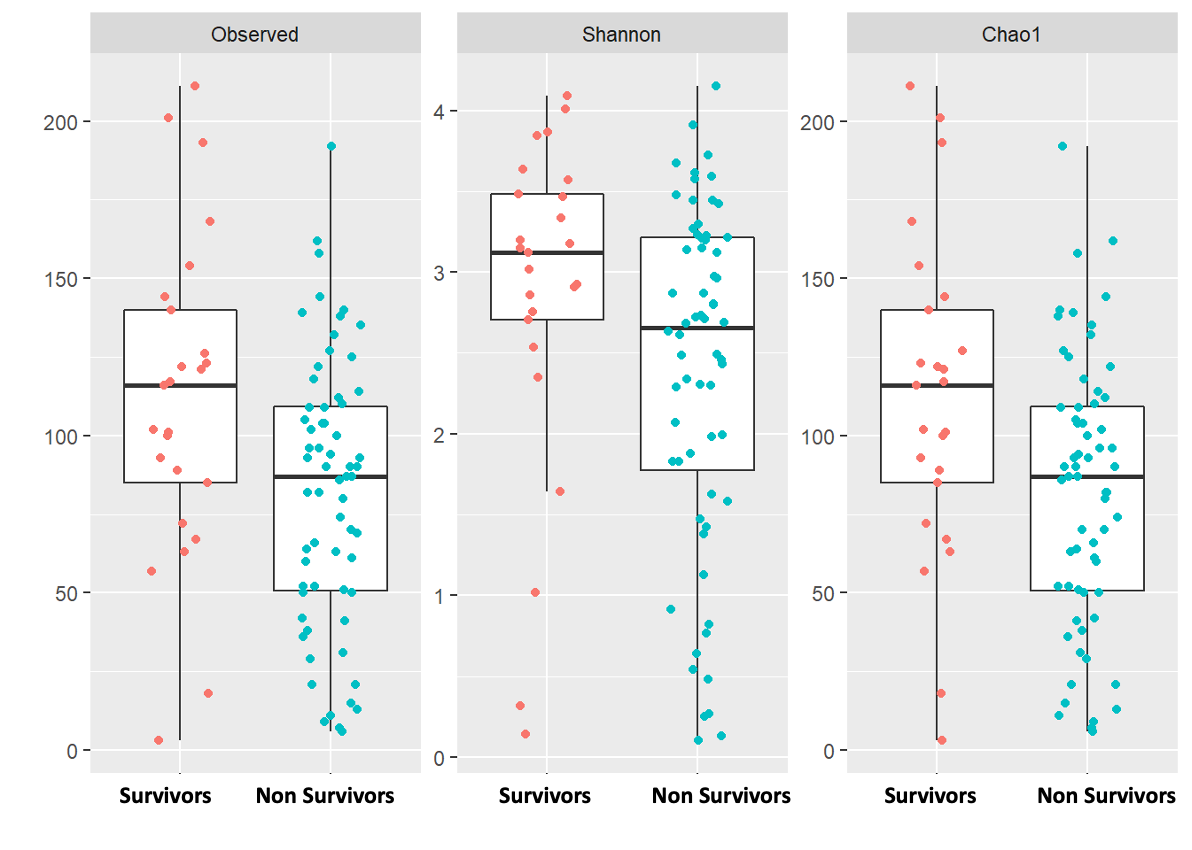


**Figure S6**. **Impact of mortality of patients with septic shock on alpha diversity of gut microbiota.** Boxes represent the IQR between the first and third quartiles; the horizontal line represents the median. Statistical significance was tested using the Mann-Whitney-Wilcoxon test. Community richness and diversity were estimated by Chao-1 and Shannon scores, respectively.


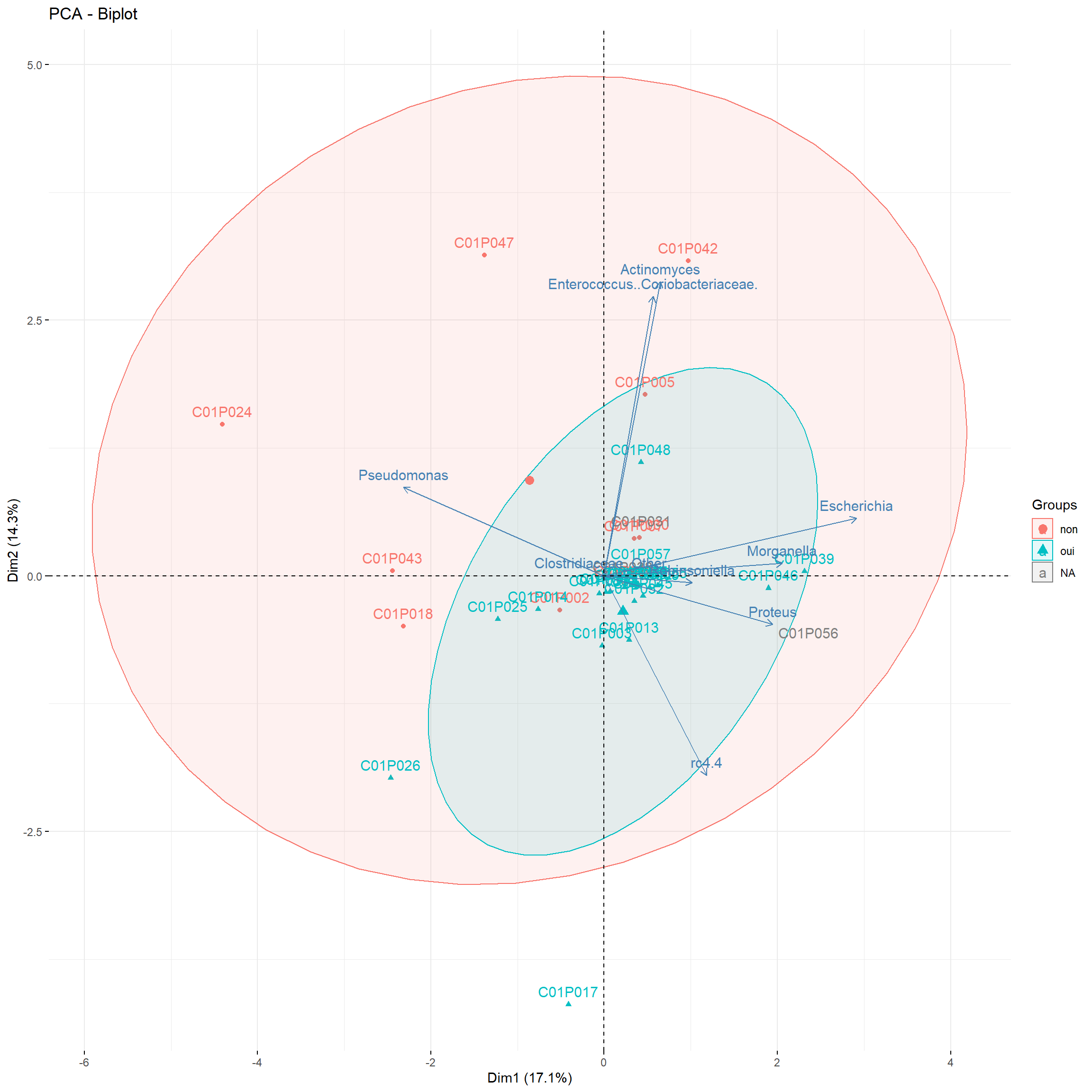


**Figure S7. Principal component analysis** **based on relative abundance of bacterial genera (those showing a statistically significant change**) **and the mortality of patients with the septic shock**. Each data point represents an individual sample. Color is indicative of the groups (survivors in blue, non-survivors in red).

# Supplementary Tables

**Table S1**. Phylum relative abundances in the gut microbiota of septic shock patients between Day 0 and Day 7.

| **Phylum** | **Relative abundance %** | | **p-value** |
| --- | --- | --- | --- |
|  | **Day 0** | **Day 7** |  |
| Firmicutes | 72.58 (±23.17) | 79.61 (±21.93) | **0.043** |
| Bacteroidota | 5.96 (±9.95) | 9.80 (±15.82) | 0.92 |
| Proteobacteria | 12.21 (±18.57) | 4.18 (±10.85) | **0.0009** |
| Actinobacteriota | 8.88 (±14.81) | 5.90 (±11.96) | **0.013** |
| Synergistota | 0.17 (±0.72) | 0.16 (±0.63) | 0.652 |
| Euryarchaeota | 0.02 (±0.06) | 0.06 (±0.16) | 0.275 |
| Verrucomicrobiota | 0.16 (±0.43) | 0.06 (±0.24) | 0.456 |
| Mycoplasmatota | 0.01 (±0.05) | 0.21 (±1.33) | 0.834 |
| Lentisphaerota | 0.00 (±0.01) | 0.00 (±0.002) | 0.756 |
| Fusobacteriota | 0.00 (±0.02) | 0.00 (±0.02) | 0.494 |
| Other | 0.00 (0.00) | 0.01 (±0.07) | 0.255 |

Statistics were performed using the Wilcoxon test. Bold values represent statistically significant differences (p<0.05). Results are expressed by mean and standard deviation (SD).

**Table S2**. Relative abundances of the main genera in the gut microbiota of septic shock patients between Day 0 and Day 7.

| **Genera** | **Relative abundance %** | | **p-value** |
| --- | --- | --- | --- |
|  | **Day 0** | **Day 7** |  |
| *Bifidobacterium* | 8.04 (±14.84) | 5.22 (±11.76) | **0.041** |
| *Parabacteroides* | 0.00 (±0.02) | 0.02 (±0.04) | 0.019 |
| *Alistipes* | 0.03 (±0.10) | 0.13 (±0.34) | **0.045** |
| *Finegoldia* | 0.64 (±2.61) | 0.26 (±1.14) | **0.023** |
| *X1855D* | 0.11 (±0.27) | 0.00 (±0.00) | <0.0001 |
| *Peptoniphilus* | 0.21 (±1.13) | 0.01 (±0.03) | 0.011 |
| *Anaerococcus* | 0.05 (±0.17) | 0.01 (±0.05) | 0.035 |
| *Christensenella* | 0.05 (±0.27) | 0.09 (±0.24) | 0.008 |
| *Sarcina* | 0.43 (±2.34) | 0.00 (±0.00) | 0.049 |
| *Anaerofustis* | 0.01 (±0.03) | 0.00 (±0.00) | 0.031 |
| *Blautia* | 5.60 (±6.44) | 2.15 (±3.5) | <0.0001 |
| *Ruminococcus* | 5.81 (±11.19) | 2.41 (±3.31) | 0.008 |
| *Lachnospiraceae* | 0.11 (±0.34) | 0.02 (±0.06) | 0.002 |
| *Coprococcus* | 4.80 (±6.22) | 3.83 (±6.84) | 0.007 |
| *Clostridium* | 1.93 (±2.96) | 1.06 (±3.31) | 0.001 |
| *Dorea* | 1.32 (±2.30) | 0.74 (±1.05) | 0.023 |
| *Roseburia* | 1.82 (±4.27) | 0.86 (±2.88) | 0.0002 |
| *Butyricicoccus* | 0.71 (±1.1) | 0.31 (±0.69) | 0.0002 |
| *Dialister* | 0.84 (±2.06) | 0.58 (±2.36) | 0.008 |
| *Staphylococcus* | 0.00 (±0.00) | 0.22 (±0.99) | 0.003 |
| *Enterococcus* | 3.28 (±8.77) | 24.90 (±34.05) | <0.0001 |
| *Escherichia* | 10.21 (±17.72) | 0.84 (±1.43) | 0.004 |
| *Shigella* | 0.03 (±0.05) | 0.00 (±0.01) | 0.001 |
| Other | 0.10 (0.30) | 0.00 (±0.02) | 0.002 |

Statistics were performed using the Wilcoxon test. Bold values represent statistically significant differences (p<0.05). Only genera with statistically significant evolution are presented. Results are expressed by mean and standard deviation (SD).

**Table S3**. Association between clinical parameters and genera detected in gut microbiota at Day 0 and Day 7 in septic shock patients.

| **Acute gastrointestinal injury (AGI) grade** |  | **AGI**  **grade I** | **AGI**  **grade II** | **AGI grade III-IV** | **p-value** | **q-value** |
| --- | --- | --- | --- | --- | --- | --- |
| **Day 0** | *Cloacibacillus* | 0.15±0.27 | 0.02±0.15 | 0.00±0.00 | 0.003 | 0.713 |
|  | *Oisenella* | 0.01±0.02 | 0.00±0.00 | 0.00±0.00 | 0.017 | 1 |
|  | *Parabacteroides* | 0.03±0.07 | 0.00±0.00 | 0.00±0.00 | 0.017 | 1 |
|  | *Gallicola* | 0.00±0.00 | 0.00±0.00 | 0.01±0.02 | 0.022 | 1 |
| **Day 7** | *Butyricicoccus* | -0.24±1.34 | -0.09±0.15 | -0.43±1.14 | 0.0066 | 0.859 |
|  | *Klebsiella* | -0.52±1.55 | 0.28±0.30 | -0.09±0.27 | 0.0126 | 1 |
| **Evolution D0-D7** | Lachnospiraceae | 0.08±0.08 | -0.13±0.36 | -0.47±0.85 | 0.0100 | 1 |
|  | *Blautia* | 4.00±7.16 | -4.07±7.81 | -5.71±4.05 | 0.0198 | 1 |
|  | *Ruminococcus* | 2.41±2.05 | -1.05±6.08 | -4.11±4.23 | 0.0199 | 1 |
|  | *Dialister* | 2.52±3.43 | -0.54±2.59 | -0.73±1.78 | 0.0267 | 1 |
|  | *Oisenella* | -0.01±0.03 | 0.00±0.02 | 0.00±0.02 | 0.0296 | 1 |
|  | *Anaerostipes* | 0.08±0.14 | -0.07±0.25 | -1.62±3.65 | 0.0357 | 1 |
|  | *Butyricicoccus* | 0.70±1.14 | -0.63±1.38 | -0.25±0.25 | 0.0389 | 1 |
|  | *Dorea* | 0.22±0.52 | -0.85±4.01 | -1.16±1.11 | 0.0392 | 1 |
| IGS II score |  | **IGS II Low** | **Moderate** | **Severe** | **p-value** | **q-value** |
| **Day 0** | *Gardnerella* | 0.00±0.00 | 0.00±0.00 | 0.03±0.07 | 0.0324 | 1 |
|  | *Clostridium* | 0.00±0.00 | 0.00±0.00 | 0.01±0.02 | 0.0324 | 1 |
|  | *Collinsella* | 0.01±0.03 | 0.04±0.08 | 0.14±0.16 | 0.0355 | 1 |
| **Day 7** | *Enterococcus* | 12.58±30.08 | 31.81±35.22 | 26.29±35.9 | 0.0133 | 1 |
|  | *Anaerotruncus* | 0.02±0.05 | 0.40±0.72 | 0.00±0.00 | 0.0169 | 1 |
|  | *Akkermansia* | 0.00±0.00 | 0.11±0.32 | 0.00±0.00 | 0.0297 | 1 |
|  | *Alistipes* | 0.06±0.19 | 0.20±0.44 | 0.02±0.04 | 0.0393 | 1 |
|  | *Roseburia* | 1.26±3.21 | 0.73±3.12 | 0.46±0.60 | 0.0434 | 1 |
| SOFA score |  | **SOFA Low** | **Moderate** | **Severe** | **p-value** | **q-value** |
| **Day 0** | *Akkermansia* | 0.14±0.45 | 0.11±0.37 | 0.42±0.52 | 0.0070 | 0.9254 |
|  | *Blautia* | 4.10±4.39 | 5.03±6.65 | 12.04±7.18 | 0.0276 | 1 |
|  | *Comamonas* | 0.00±0.00 | 0.00±0.00 | 0.04±0.11 | 0.0324 | 1 |
|  | *Garnerella* | 0.00±0.00 | 0.00±0.00 | 0.03±0.07 | 0.0324 | 1 |
|  | *Faecalibacterium* | 0.00±0.00 | 0.00±0.00 | 0.01±0.02 | 0.0324 | 1 |
|  | *Butyrivibrio* | 0.00±0.00 | 0.00±0.00 | 0.07±0.18 | 0.0324 | 1 |
|  | *Parabacteroides* | 0.00±0.00 | 0.00±0.00 | 0.03±0.07 | 0.0324 | 1 |
|  | *Eggerthella* | 0.24±0.36 | 0.21±0.32 | 0.01±0.02 | 0.0424 | 1 |
|  | *Alloscardovia* | 0.00±0.00 | 0.05±0.24 | 0.11±0.20 | 0.0478 | 1 |
| **Day 7** | *Peptococcaceae* | 0.00±0.00 | 0.05±0.10 | 0.06±0.05 | 0.0098 | 1 |
|  | *Proteus* | 0.00±0.00 | 0.00±0.00 | 0.38±0.76 | 0.0098 | 1 |
|  | *Blautia* | 1.32±2.85 | 1.83±2.52 | 7.15±6.68 | 0.0342 | 1 |
| **Evolution D0-D7** | *Garnerella* | 0.00±0.00 | 0.00±0.00 | -0.05±0.10 | 0.0125 | 1 |
|  | *Clostridiaceae* | -0.07±0.16 | 0.01±0.07 | 0.35±0.41 | 0.0251 | 1 |
|  | *Akkermansia* | -0.01±0.27 | -0.08±0.55 | -0.54±0.59 | 0.0335 | 1 |

All data are expressed by mean ±standard deviation. Only genera with statistically significant evolution are presented.

**Table S4**. Association between the outcome of patients (survivor or non-survivor at day 28) and genera detected in gut microbiota at Day 0 and Day 7 in septic shock patients.

|  |  | **Survivor at D28** | **Non-survivor at D28** | **p-value** | **q-value** |
| --- | --- | --- | --- | --- | --- |
| **Day 0** | *Mogibacteriaceae* | 0.59±0.84 | 1.42±1.53 | 0.0146 | 1 |
|  | *Robinsoniella* | 0.00±0.00 | 0.02±0.04 | 0.0219 | 1 |
|  | *Klebsiella* | 0.19±0.81 | 0.58±1.24 | 0.0269 | 1 |
|  | *Proteus* | 0.01±0.04 | 0.08±0.21 | 0.0281 | 1 |
| **Day 7** | *Enterococcus* | 0.00±0.02 | 0.05±0.09 | 0.0085 | 0.992 |
|  | *Roseburia* | 0.61±2.27 | 0.37±0.48 | 0.0097 | 1 |
|  | *Eggerthella* | 0.21±0.86 | 0.83±1.98 | 0.0206 | 1 |
|  | *Eubacterium* | 0.14±0.32 | 0.42±0.87 | 0.0216 | 1 |
|  | *Victivallaceae* | 0.00±0.00 | 0.01±0.03 | 0.0275 | 1 |
|  | *Pseudomonas* | 0.00±0.00 | 5.81±19.09 | 0.0275 | 1 |
|  | *Gemellaceae* | 0.00±0.01 | 0.03±0.05 | 0.0296 | 1 |
|  | *Christensenellaceae* | 0.75±1.85 | 2.67±3.50 | 0.0365 | 1 |
|  | *Actinomyces* | 0.01±0.03 | 0.04±0.07 | 0.0378 | 1 |
| **Evolution D0-D7** | *Enterococcus* | 0.00±0.02 | 0.05±0.08 | 0.0071 | 1 |
|  | *Actinomyces* | -0.13±0.41 | 0.03±0.07 | 0.0153 | 1 |
|  | *Proteus* | 0.00±0.02 | -0.11±0.25 | 0.0046 | 1 |
|  | *Pseudomonas* | 0.00±0.01 | 6.39±20.02 | 0.0298 | 1 |
|  | *Escherichia* | -4.66±11.78 | -9.21±10.82 | 0.0303 | 1 |
|  | *Clostridiaceae* | -0.02±0.19 | 0.04±0.06 | 0.0464 | 1 |

All data are expressed by mean ±standard deviation. Only genera with statistically significant evolution are presented.
